# A multi-faceted role of dual-state dopamine signaling in working memory, attentional control, and intelligence

**DOI:** 10.3389/fnbeh.2023.1060786

**Published:** 2023-02-16

**Authors:** Louis D. Matzel, Bruno Sauce

**Affiliations:** ^1^Department of Psychology, Rutgers University, Piscataway, NJ, United States; ^2^Department of Biological Psychology, Vrije Universiteit Amsterdam, Amsterdam, Netherlands

**Keywords:** dopamine, intelligence, working memory, attention, receptor subtypes, behavioral genetics

## Abstract

Genetic evidence strongly suggests that individual differences in intelligence will not be reducible to a single dominant cause. However, *some* of those variations/changes may be traced to tractable, cohesive mechanisms. One such mechanism may be the balance of dopamine D1 (D_1_R) and D2 (D_2_R) receptors, which regulate intrinsic currents and synaptic transmission in frontal cortical regions. Here, we review evidence from human, animal, and computational studies that suggest that this balance (in density, activity state, and/or availability) is critical to the implementation of executive functions such as attention and working memory, both of which are principal contributors to variations in intelligence. D1 receptors dominate neural responding during stable periods of short-term memory maintenance (requiring attentional focus), while D2 receptors play a more specific role during periods of instability such as changing environmental or memory states (requiring attentional disengagement). Here we bridge these observations with known properties of human intelligence. Starting from theories of intelligence that place executive functions (e.g., working memory and attentional control) at its center, we propose that dual-state dopamine signaling might be a causal contributor to at least some of the variation in intelligence across individuals and its change by experiences/training. Although it is unlikely that such a mechanism can account for more than a modest portion of the total variance in intelligence, our proposal is consistent with an array of available evidence and has a high degree of explanatory value. We suggest future directions and specific empirical tests that can further elucidate these relationships.

## Introduction

Performance across cognitive tasks co-varies, e.g., people good at navigating a map are usually good at doing math, remembering a shopping list, writing an essay, learning quickly, and analyzing the logic of an argument. This pattern, referred to as the “positive manifold,” is one of the most well-replicated observations in human psychology (Plomin and Spinath, 2002). The positive manifold makes it possible to distinguish people’s cognitive abilities based on a single aggregate index, forming the logic behind quantitative tests of intelligence, i.e., IQ tests. Large differences in IQ scores are immediately obvious to even the most casual observer, and quantitatively, performance on an IQ test is a strong predictor of academic, career, and social success (Gottfredson, 1998), as well as a multitude of other life outcomes such as mortality, drug addiction, criminal behavior, and creativity (Schmidt and Hunter, 1998; Johnson et al., 2009; Wilson et al., 2009; Benedek et al., 2014).

The underlying basis for individual differences in intelligence has been difficult to elucidate, and the ultimate solution is likely to be more complicated and multi-faceted than is often assumed. Intelligence has an (in)famously high heritability, where in some populations/contexts, it can be as high as 60–70%, meaning that of all individual differences in intelligence, 60–70% are explained by genetic differences among people. However, specific (additive) candidate genes and DNA regions seem to explain less than 0.01% of the total variance in intelligence (Chabris et al., 2012). And large-scale genome-wide association studies (GWASs) have revealed that even a combination of thousands of (additive) DNA regions accounts for only 10–20% of cognitive differences (Sniekers et al., 2017; Lee et al., 2018; Okbay et al., 2022). But what about mechanisms related to our environments? Even the longest and most widespread cognitive experience in our lives (i.e., schooling) has a limited (additive) impact on intelligence, promoting an average increase of 0.2 standard deviations per year (Ritchie and Tucker-Drob, 2018). Likewise, a large study in the UK suggests that differences in education quality between standard schools and highly selective, prestigious schools account for less than 1% of the variation in national exam scores (Smith-Woolley et al., 2018). The impact of other experiences on intelligence, from physical exercise and nutrition to digital media and computerized cognitive training seems to be even more modest (when present at all).

Based on the previous evidence, it is virtually certain that variations in intelligence will not be fully explained by “single” life experiences, a small network of genes, a single brain region, or a simple neural mechanism (Engelhardt et al., 2019; Matthews and Turkheimer, 2021; Deary et al., 2022). In fact, the genetic and environmental evidence paint a gloomy picture for any search for tractable, cohesive mechanisms to describe the basis for variations in intelligence. If the impact of single genes or experiences on the differences/changes in intelligence is always tiny, then causal networks will always have hundreds or even thousands of moving parts – too many for any tractable or intelligible scientific model. However, effects needn’t always be so tiny. Most identified influences on variations/changes in intelligence are additive, i.e., not sensitive to gene-environment correlations (like when small genetic differences evoke specific cognitive intermediation from teachers or parents), gene-environment interactions (when an impact is more than the sum of the genetic and environmental parts), and gene-gene interactions (networks of genes). There is evidence to support the contention that intelligence is a trait with unusual amounts of gene-environment correlations and interactions (Dickens and Flynn, 2001; Tucker-Drob et al., 2013; Sauce and Matzel, 2018; Selzam et al., 2019). Important to our point here, such gene-environment interplays are likely to dilute additive or independent genetic effects because it’s hard to identify the specific, predisposing environments or responses to or from the environment, because the genetic variation examined has little effect outside that environment, and because the analyses would require enormous samples (Manuck and McCaffery, 2014; Sauce and Matzel, 2018). So despite being difficult to detect, bigger effects might exist for intelligence and the picture might not be as gloomy and intractable as available data might initially suggest.^1^ To add optimism, we know this is already the case in some traits for non-human animals and plants, and studies considering gene-environment and gene-gene interactions have led to the development of promising tractable, intelligible causal models (Burns et al., 2012; Li et al., 2018).

Regarding the etiology of intelligence, it could be said that “many roads lead to Rome.” Many of those roads are going to be long, labyrinthine, full of gaps, and crossing multiple provinces. But a few might be shorter and well-lit. It would be valuable to identify one of those more viable roads, thereby allowing us to develop specific hypotheses and potential intervention strategies. Here, our goal is to suggest one such mechanism that is consistent with a wealth of empirical evidence: the dual state process of dopamine activity, its role in working memory/attention, and its potential as a causal contributor to *some* variation/changes in intelligence.

### The structure of general cognitive abilities

Before we describe the potential role of dual-state dopamine signaling in the expression of intelligence, we should make explicit some assumptions about the structure of cognitive abilities. The existence of the positive manifold leaves open multiple possibilities about what intelligence is and how it’s structured. For example, it could be that various cognitive domains interact during development (feeding each other and gradually giving rise to positive correlations). Alternatively, various cognitive domains could all be causally influenced by a top-down process, or it could be that cognitive tasks overlap and commonly sample the same elementary functions (giving rise to positive correlations “by accident”). Although these theories get rather complicated and there are still multiple viable contenders (Kovacs and Conway, 2016; Savi et al., 2019; Protzko and Colom, 2021), here we will assume that theories that place *working memory* and its reliance on *executive attention* at their center, either as a top-down process (such as in Hierarchical Theories) or as the elementary process that overlaps among tasks (such as in the Process Overlap Theory; (Kovacs and Conway, 2016; Burgoyne et al., 2022) provide the best framework from which to assess tractable brain mechanisms for intelligence. As dopaminergic activity plays a critical role in the implementation of the working memory system, this is not only theoretically justified, but it also provides a convenient point of contact between dual-state dopamine signaling and intelligence.

Numerous studies have supported the contention that the efficacy of the working memory system accounts for as much as 50–60% of the variability in intelligence across individuals (e.g., Conway and Engle, 1996; Engle et al., 1999; Sub et al., 2002; Colom et al., 2004; Ackerman et al., 2005), and this relationship is a central feature of broadly accepted neuroanatomical systems-level descriptions of intelligence (Jung and Haier, 2007; Colom et al., 2010; Tang et al., 2010; Bowren et al., 2020). While a great deal of attention has been directed at the elucidation of the relevant neuroanatomical networks, much less progress has been made in describing the cellular and subcellular dynamics that give rise to these network-level interactions. Understanding complex cognitive processes cannot come solely from analyses of network-level activity but will require a full description of these “micro” level events (Paquola et al., 2022).

Before attempting to describe the cellular basis for intelligence, it will be necessary to first provide a more detailed description of the working memory system and its role in intelligence.

### The working memory/attentional system

Working memory is sometimes confused with simple short-term memory, and this misconception (particularly in studies of laboratory animals) makes it difficult to appreciate the full impact of working memory on cognitive processes. According to Harrison et al. (2015), “working memory includes a *system* [emphasis ours] of temporary memory stores, the functions of retrieval and maintenance into and out of those stores, and the executive attention necessary for the performance of these functions.” One of the most clear examples of the distinction between working memory and simple short-term memory is a seminal experiment by Daneman and Carpenter (1980). In that study, human subjects were asked to remember a list of words (simple short-term memory) or to remember the same words when they were presented at the end of sentences that formed a paragraph. While simply remembering the list of words was easy (most normal humans can remember 6–8 words without error; Miller, 1956), remembering the same list of words while simultaneously reading and trying to comprehend a paragraph is exceedingly difficult, and humans will both forget words on the list and respond with incorrect words that were not on the list. This decline in performance is a product of *interference*, i.e., reading comprehension is cognitively demanding and competes with the capacity to simultaneously store information for later retrieval. What was particularly interesting about Daneman and Carpenter’s (1980) observations was that the capacity of simple short-term memory had little relationship to reading comprehension (measured separately). However, the ability to remember words while simultaneously processing information (i.e., comprehending) was a strong predictor of more general reading comprehension (which, incidentally, is a close proxy for intelligence as captured on an IQ test). This provides a clear example of the distinction between simple short-term memory (or “simple span”) and working memory span (or “complex span”). Thus, while working memory is dependent on short-term memory, it is a far broader cognitive skill, utilizing not only memory, but attention, resistance to distraction, and goal direction.

### The contribution of working memory/attention to variations in intelligence

Given the breadth and impact of the working memory system, it is not surprising that it is often asserted that working memory is engaged by virtually *all* cognitive tasks, qualifying it as a “core” cognitive ability (Engle, 2018). The conceptual relationship between working memory and general cognitive functioning is validated by the consistent observation that working memory capacity (but often not simple short-term memory; see Martinez et al., 2011, for an alternative view) is strongly correlated with performance on standard intelligence (IQ) tests in humans (Conway et al., 2003; Unsworth and Engle, 2006, 2007; Harrison et al., 2015) and laboratory animals (Kolata et al., 2005, 2007, 2008; Burgoyne et al., 2020).

As noted above, working memory is not a single cognitive function, but rather a collection of cognitive functions that work in unison, engaging short-term memory, attention/resistance to interference, and goal tracking/updating. Despite the strong and consistent relationship between tests of composite working memory capacity and intelligence, it is important to recognize that all of these independent functions do not correlate equally (or contribute equally) to variations in intelligence. As noted above, Daneman and Carpenter (1980) reported that simple short-term memory was only weakly related to reading comprehension (a proxy for intelligence), while working memory capacity (c.f., “complex span”) was a strong predictor. This exemplifies the fact that while a system devoted to the *storage* of information is critical to the function of working memory, other aspects of working memory, particularly those representative of executive functioning (and specifically dependent on the prefrontal cortex; PFC^2^), are more critical to the instantiation of variations in intelligence. Recent evidence suggests that attention and/or attentional disengagement (necessary for task-switching or updating) may play a particularly important role in the relationship between the working memory system and intelligence (Engle, 2002, 2018; Kolata et al., 2007; Sauce et al., 2014; Burgoyne and Engle, 2020; Crawford et al., 2020; Li and Cowan, 2022).

Notably, experimental studies of attentional disengagement (or the ability to ignore previously relevant information that is now obsolete) are based on dramatic changes in established “memories” or behavioral tendencies. However, attentional shifting can be more subtle and pervasive in nature and can contribute to performance on otherwise trivial cognitive tasks, as well as tasks that are represented in IQ tests. For instance, during performance on the Raven’s Progressive Matrix IQ test, an individual must store (in memory) partial solutions to a problem, assess the utility of those solutions, then either progress to further tests of the solution, or reject the solution. In the latter case, a new solution must be devised, and the old (incorrect solution) must be purged from active memory so as not to interfere with tests of the new solution (for a more detailed description of these processes, see Carpenter et al., 1990).

### DA’s role in the regulation of working memory/attention/intelligence

Having established that the efficacy of the working memory system is a strong determinant of intelligence, we now turn to the role of DA in the regulation of working memory and its impact on the expression of intelligence. Dopamine in the prefrontal cortex (PFC) may play two roles in the regulation of the efficacy of working memory/attention. First, DA can modulate the gain of relevant classes of neurons, e.g., PFC pyramidal neurons can respond to DA by increasing the single neuron gain such that signal-to-noise ratios are optimized for an increase in perceptual sensitivity (Li et al., 2006; Sikstrom, 2007). Secondly, DA may support the co-existence of two stable states in single pyramidal neurons, i.e., a resting state and sustained activity. This bi-stability has been suggested to act as a neural foundation for the instantiation of working memory (or more precisely, short-term memory) through the active maintenance of signals (e.g., perceptual input or recalled memories) during periods of maintenance (Goldman-Rakic, 1995; Durstewitz et al., 2000a). In computational models, this latter function of DA has been shown to stabilize the maintenance of information (*via* an increase in the signal-to-noise ratio) such that it is less subject to interference during the retention of currently relevant information (Durstewitz et al., 2000b). Further, Brunel and Wang (2001) demonstrated that the supplementation of DA stabilized activity in a network of cells such that they were resistant to both synaptic “noise” and distracting perceptual stimuli. In combination, these effects (increased gain and bi-stability) can strengthen the representation of signals and reduce the interference imposed on information intended for short-term maintenance (also see Servan-Schreiber et al., 1998, for explicit descriptions of the role of DA in focused attention). This hypothesis has found broad experimental support in studies both of laboratory animals (Seamans et al., 2001; Lavin et al., 2005) and humans (Muller et al., 1998; Tost et al., 2006). As described above, there has been a recent emphasis on the role of the efficacy of information updating i.e., a lack of dysfunctional attentive perseveration, as a critical component of the relationship between working memory and intelligence. Working memory requires not only the efficient storage and updating (addition and deletion) of information but also the ability to ignore *previously* relevant information. Absent this ability, the subject cannot efficiently adapt to new or changing conditions (a prerequisite for a multitude of cognitive functions; Carpenter et al., 1990; Engle, 2018). As described in the previous paragraph, DA (and in particular, DA’s effects on excitation *via* the D_1_ receptor) may play a central role in the maintenance of information. However, DA effects on the D_2_R are generally antagonistic to the D_1_R in prefrontal areas (Gulledge and Jaffe, 1998; West and Grace, 2002). This competition between the two receptor classes may favor the D_2_R during changing conditions or task-shifting, thus promoting cognitive flexibility. Given this possibility, we should ask whether there is a neurophysiological basis by which environmental/cognitive states could regulate this balance. In this regard, it appears that D1 and D2 receptors are specifically sensitive to DA concentrations, where D2 receptors preferentially respond to low levels of DA, and D1 receptors respond preferentially to higher levels. Moreover, while the D2 receptor may be activated first in response to dopaminergic input, the D1 receptor may dominate later responses under stable conditions (Lapish et al., 2007; Schultz, 2007). These observations have led to the suggestion by Durstewitz (2006) and Durstewitz and Seamans (2006) that the state regulated by D1 and D2 receptors might be determined by task-dependent release of DA, and that the temporal order of states is such that the D2 receptor could dominate during the emergence of new responses, whereas the D1 receptor could dominate stable or established responses. It is worth noting that such a context-dependent mechanism of attentional regulation *could be* a consequence of a type of gene-environment interplay, i.e., different people can have D1 and D2 profile levels and these can be amplified or reduced depending on the cognitive environment.

Evidence for a dual-state of DA function in the regulation of working memory/attention comes from both experimental (Wang et al., 2004; Floresco and Magyar, 2006) and computational studies (Durstewitz and Deco, 2008; Durstewitz and Seamans, 2008; Rolls et al., 2008). These two classes of studies each suggest that the ratio between D1 and D2 receptor availability or activation may determine the balance between maintenance of information and flexibility when conditions change. Notably, these experimental and computational studies are based on dramatic changes in established “memories” or behavioral tendencies. Recall however (as described above), attentional disengagement has a broad impact on cognitive function beyond simple laboratory tests, and can, for instance, be an important determinant of performance during problem-solving (such as represented on the RPM test of IQ).

The previous discussion suggests that insufficient *or* excessive DA could impair performance of tasks dependent on working memory (i.e., either condition could result in an imbalance of D1 and D2 receptors). In a recent analysis, Papenberg et al. (2020) examined the balance between transmitter availability and the density of D2 receptors, and found that individuals for whom D2 receptor density matched DA availability performed better on a working memory task relative to individuals with insufficient or excessive DA relative to the number of receptors. These results suggest that the inverted U–shaped function between DA levels and the efficacy of working memory is consequent to a dynamic association between DA availability and the density of receptor subtypes.

As noted, cognitive traits in humans have enormously complex origins. Consequently, it has been argued that a complete understanding of these traits will require consideration of laboratory studies of animals, in part because these model systems are subject to precise control, both of environmental and genetic variations (Robbins, 2018).

Using genetically heterogeneous mice as subjects, Kolata et al. (2010) characterized the animals’ general cognitive abilities based on their aggregate performance across a battery of diverse learning tasks. With a procedure designed to minimize false positive identifications, analysis of gene expression microarrays (comprised of ≈25,000 genes) identified a small number (<20) of genes (based on this particular selection criterion) that were differentially expressed across animals that exhibited fast or slow aggregate learning abilities. Of these genes, one functional cluster (Darpp-32, Drd1a, and Rgs9) was identified, and this cluster is an established modulator of dopamine signaling, and in particular, the relative balance of D1 and D2 activity. Subsequent quantitative PCR found that expression of these dopaminergic genes was significantly correlated with animals’ general cognitive performance.

Using mice that were characterized for general cognitive abilities similar to Kolata et al. (2010), Wass et al. (2013) injected the mice with a D1 agonist and then assessed the relationship between individuals’ general cognitive performance and activation of D1 receptor- containing neurons in the prelimbic region of the medial prefrontal cortex, the agranular insular cortex, and the dorsomedial striatum. Increased activation of D_1_R -containing neurons in the prelimbic cortex (but not the agranular insular cortex or dorsomedial striatum) was observed in animals characterized as more “intelligent” relative to less intelligent cohorts. Given the results of Kolata et al. (2010) and these results of Wass et al. (2013), it was reasonable to anticipate that the density of D_1_R would be directly related to general cognitive performance. However, no differences in the *density* of D_1_Rs could be detected in the cell membranes of animals of high vs. low abilities. This seeming paradox was resolved in another experiment by Wass et al. (2018). Mice were administered an irreversible dopamine receptor antagonist (EEDQ), after which the density of newly inserted D1 receptors was quantified, and it was determined that general cognitive abilities were positively correlated with the rate of D1 receptor recovery. This observation suggests that a sub-membrane level influence on the trafficking of immature (sequestered) D1 receptors differentially regulates the *availability* of these receptors across animals that exhibit variations in general cognitive performance. This hypothesis suggests that a pool of D1 receptors may be available to meet the demands of cognitive challenges and that the availability of immature receptors for transport to the plasma membrane may contribute to variations in intelligence. It is also notable that newly inserted receptors are more sensitive to bound transmitter than are older receptors (Ferguson, 2001; Greenbaum et al., 2003), suggesting that even when receptor density is equivalent, receptors in high IQ individuals might be more responsive.

The dopamine receptor interacting protein, DRiP78, regulates the export of immature receptors from the ER to the Golgi complex and their subsequent trafficking to the plasma membrane. DRiP78 binds to the triple phenylalanine motif [F(X)3F(X)3F] on the C-terminus of the D1 receptor (and is thus believed specific to this receptor), and mutations of the three phenylalanines result in a complete loss of receptor localization on the plasma membrane of dopaminergic neurons (Bermak et al., 2001). A tightly controlled balance of DRiP78 is required for efficient trafficking of the receptor to the plasma membrane, and elevated levels of DRiP78 promote the sequestration of immature D1 receptors in the ER (Bermak et al., 2001). We can then hypothesize that under physiological/cognitive resting conditions, animals exhibiting higher cognitive abilities would express relatively higher levels of DRiP78, thus maintaining a larger intracellular pool of receptors awaiting recruitment to the plasma membrane, accompanied by an increased rate of receptor turnover consequent to periods of high cognitive demands. Indeed, Wass et al. (2018) have reported that general cognitive abilities were positively correlated with both the rate of D1 receptor recovery and levels of DRiP78. These results provide evidence that innate general cognitive abilities are related to D1 receptor turnover rates and the pool of available receptors in the prefrontal cortex, such that an intracellular pool of immature D1 receptors are available to potentially accommodate cognitive demands.

The results of Wass et al. (2018) are particularly revealing regarding the nature of variations in intelligence. It is established that in humans, attentional “fatigue” (experimentally induced) promotes a decline in cognitive performance (Csatho et al., 2012). After these induced declines in attentional performance, the correlation between measures of reaction time and IQ increases significantly, suggesting that low IQ individuals are less prepared to respond to attentional fatigue than are high IQ individuals (Larson and Alderton, 1990; for implications, see Kovacs and Conway, 2016). Wass et al.’s (2013) results suggest that high-IQ individuals might have a greater reserve of dopamine receptors that are available to respond to high cognitive demands, thus placing the high-IQ individual at an advantage when faced with complex cognitive challenges. In support of this hypothesis, Wass et al. (2018) reported that the density of D1 receptors increased within <60 min (although possibly much more rapidly) in response to intense demands on working memory, suggesting that a pool of immature receptors was available to accommodate high cognitive loads.

The above results speak to the contribution of the Dopamine D1 receptor to efficient cognitive performance as it relates to an individual’s “intelligence.” As described above, the role of the D2 receptor might be best observed in tasks explicitly designed to isolate cognitive flexibility or task-shifting, as cognitive flexibility has been hypothesized to be neurophysiologically tied to properties of the D2 receptor during dopaminergic transmission. With this as their goal, Zmigrod and Robbins (2021) assessed the performance of 1,400 humans’ on the Wisconsin Card Sorting Task (WCST), a task in which previously learned rules for card sorting can interfere with an individual’s performance when the rules for sorting are updated (e.g., when switched from “sort by color” to “sort by number”). The WCST is known to be particularly sensitive to age-related cognitive declines, a hallmark of which is debilitating perseveration consequent to a loss of cognitive flexibility (Rhodes, 2004; Head et al., 2009). Zmigrod and Robbins (2021) found single nucleotide polymorphisms associated with elevated DA levels in the PFC predicted performance on this task. Importantly, gene-gene interactions were observed between catechol-O-methyltransferase (which contributes to the degradation of DA) and the D2 receptor, such that genotypes with elevated prefrontal DA performed best during rule changes, suggesting that levels of the D2 receptor were associated with cognitive flexibility.

### Modulation of DA dynamics by experience and cognitive demands

As noted above, using mice as subjects, Wass et al. (2013) reported that intense demands on working memory could induce an increase in the expression of D1 receptors in the PFC, and moreover, Wass et al. (2018) found that D1 receptors became rapidly available during periods of high cognitive demand. Moreover, availability was positively correlated with animals’ general cognitive ability. These results suggest that a pool of D1 receptors may be available to meet the demands of cognitive challenges and that the availability of immature receptors for transport to the plasma membrane may contribute to variations in intelligence. Such a mechanism might broadly mediate the beneficial effects of “cognitive exercise,” or more generally, cognitive experience (e.g., education or adoption to higher SES environments).

At best, experimentally imposed cognitive exercise has small and transient benefits on traits like intelligence. While broad experiences (e.g., adoption from low into high SES environments) and changes in economic status (and the cognitive opportunities that it affords) *can* promote dramatic improvements in intelligence (Schiff et al., 1982; Rutter, 1998; Rindermann and Thompson, 2016; for review, see Sauce and Matzel, 2018), relatively minor laboratory manipulations have met with very limited success (c.f., Redick et al., 2012). In the limited cases of typical laboratory manipulations, a few weeks of cognitive “exercise” is unlikely to enhance intelligence, although there have been solid, large-sample studies that have indeed found an enhancement of working memory, attention, and intelligence – sometimes lasting over 1 year (Judd and Klingberg, 2021).

Improvements in working memory/attention might be in part a product of the increased availability of D1 receptors in the PFC. Such an effect was observed in human subjects by McNab et al. (2009). Human adults were exposed to progressively more difficult working memory training for 5 weeks, and D1 receptor binding potential was observed by MRI before and after this training. Five cortical regions were activated by the working memory training itself, and increased binding to the D1 receptor was observed in the PFC as a function of this training. A similar pattern of results in children and adolescents has been reported by Söderqvist et al. (2014). These results demonstrate that practice-induced changes in working memory correlate with changes in transmission at the D1 receptor, confirming that in humans, the D1 receptor *can* be modulated in response to cognitive demands.

### A general role of dopamine in higher cognitive abilities: Signal prediction error and computational learning

Intelligence (such as indicated by an IQ test) is more highly reflected in tasks that make more complex cognitive demands. This may not be surprising given the obvious assumption that increasing cognitive complexity increases demands on intelligence. While what are described as “elemental” tasks (such as simple paired associate learning) are weakly correlated with IQ (Jensen, 1985), increasing complexity increases the correlation of a cognitive task with IQ (Williams and Pearlberg, 2006). A simple explanation of this relationship is that increasing the complexity of *any* task is likely to place higher demands on working memory and attention. In the present context, we might similarly expect that “higher” cognitive tasks (that make greater demands on working memory/attention) would be ones that were most obviously dependent on dopamine.

It is commonly asserted that dopamine-responsive neurons respond preferentially to appetitive stimuli as well as reward-predicting stimuli (Berridge, 2007; Lerner et al., 2021). However, recent evidence indicates that these cells do not respond unconditionally to rewarding stimuli, but instead signal deviations from the prediction of future events, suggesting that they are involved more broadly in several higher cognitive functions. Schultz et al. (1997) have argued that these reward-related responses correspond formally to concepts of computational learning. Supporting this role, it has been observed that dopaminergic neurons in the primate can exhibit variable outputs that correlate with changes or errors in the predictions of future rewarding events (Schultz and Clark, 1997). “Prediction error” certainly requires attention (whereas simply responding to a reward may not), and it has been observed that accumbal dopamine release corresponds in a valence-independent manner to the perceived salience of stimuli, suggesting that dopamine can serve to facilitate domain-independent attentional processes (Kutlu et al., 2021).

Regarding the role of attention in variations in intelligence, Sauce et al. (2014) and Crawford et al. (2020) reported that resistance against *internal* sources of interference (e.g., previously learned behavioral responses) was a better predictor of general intelligence than was resistance against external sources of interference (e.g., environmental distractors). This was demonstrated (in part) by the observation that more intelligent animals were quicker to overcome “latent inhibition” than were less intelligent animals. Latent inhibition is the process by which animals learn to ignore stimuli that are meaningless, and thus are slow to later associate that stimulus with a reward. It was hypothesized that when conditions changed (i.e., when a previously meaningless stimulus was reinforced), more intelligent animals could more quickly adapt to the new contingency. This ability to ignore a *previously* acquired memory in favor of new conditions is emblematic of resistance against an internal source of interference. Kutlu et al. (2022) have shown that dopamine in the nucleus accumbens is evoked by novel stimuli, but that this response habituated with successive presentation of the stimulus, corresponding with a decrease in behavioral responsivity. This habituation process reduced the future ability of that stimulus to enter into an association with a reward (i.e., latent inhibition). In support of this role for dopamine, optogenetic manipulation of the dopamine responses to a cue could bi-directionally influence the capacity of a stimulus to enter into an association with a reward. Kutlu et al. (2021) concluded that dopamine did not merely respond to rewarding stimuli, but rather, played a causal role in modulating variations in attention.

As we noted previously, lower IQ is associated with faster attentional fatigue, and consequently, relatively poor performance on tasks that make prolonged demands on attention. Low levels of stimulants such as methylphenidate (a dopamine re-uptake blocker) are often found to improve cognitive performance, and this has been asserted to reflect the effect of the stimulant (primarily through action in the PFC) on working memory, attention, and inhibitory control (Bagot and Kaminer, 2014; Ilieva et al., 2015; Spencer et al., 2015). One recent study provides strong support for this assumption. Westbrook et al. (2020) reported that human subjects with higher striatal dopamine synthesis were more able to expend cognitive effort. Importantly, sulpiride (a D2 receptor antagonist), increased cognitive motivation preferentially in subjects with lower synthesis capacity. These observations provide clear evidence for the competing roles of the D1 and D2 receptors on cognitive performance, possibly as a consequence of their role in the instantiation of attention.

### A DA dual-state view of variation in cognitive performance related to intelligence

Based on available evidence, it appears likely that synaptic and subcellular dopaminergic signaling is a crucial contributor to variations in working memory and attentional control, the efficacy of which contributes to variations in intelligence.

Variations in the dopaminergic dual-state system are probably highly polygenic in humans. In other words, its variation among healthy and clinical populations might be regulated by many genes, each with small effects. While the dopaminergic system seems relatively simple (with a single transmitter and two classes of receptors), genes could regulate a variety of proteins and subcellular functions such as turnover, production, transport to the membrane, transmitter release/reuptake, etc. This could explain why DNA regions surrounding dopamine genes do not show large hits in human genome-wide association studies. If so, we could expect that twin studies and genome-wide association studies done on the dual-state dopamine system (perhaps using measurements of the dual system in humans similar to Papenberg et al., 2020) will show a high genetic correlation with intelligence.

Given the current state of evidence, it is not possible to propose a specific hypothesis regarding the dopamine dual-state’s role in the instantiation of the positive manifold that describes the structure of intelligence. The working memory system (with its demands on attention) is itself a collection of overlapping cognitive functions, and even in combination, variations in the efficacy of the aggregate working memory *system* only account for a portion (∼50–60%) of the variance in intelligence. Consequently, we cannot provide a complete description of intelligence or of individual differences in that trait, but rather, are suggesting some *potential* attributes of intelligence at a cellular or molecular micro-level. Such a mechanism may explain *some* proportion of variations in intelligence, and this mechanism already shows promise in animal research and computational models.

Despite 100+ years of empirical research on intelligence and IQ, there have been few attempts to account for variations in intelligence at the cellular level. In their rare instances, such descriptions (e.g., Geary, 2018) have been met with skepticism (e.g., De Boeck and Kovacs, 2020; Matzel et al., 2020). In part, this reflects the fact that intelligence is a complex trait, with many overlapping and interacting genetic and environmental influences. Nevertheless, the general theme that emerges from the above discussion may act to bridge bridge some gaps between niches of research and may provide one target of interest. Researchers working with dopamine and the dual state theory might, for example, be interested in testing the degree of variance across humans in dopamine D1/D2 balance. Some individuals might have higher D1 signaling than others and be at different points in the inverted “U” shape curve of attentional control, and these individual differences might have, in part, genetic sources and interplay with distinct environmental experiences. Traditionally, these research domains have focused on experimental evidence of “group averages,” and thus might be insensitive to important clues regarding individual differences. Meanwhile, researchers focused on individual differences in human intelligence might be unaware of cellular-level mechanisms like the dual state, as well as with relevant animal and computational models. This later research endevour might benefit from a connection with tractible cellular mechanisms.

The question “What causes differences in intelligence?” has proven to be difficult to answer. However, given the expansive impact of intelligence on practical life outcomes, the answer will likely have broad applications to human health and well-being. The elucidation of the answers to this problem may reveal mechanisms for treating cognitive disabilities and for enhancing intelligence in our populations (and producing commensurate increase in general quality of life). This kind of progress though requires both tractable hypotheses on causal mechanisms as well as frameworks from which to assess them.

## Author contributions

LM and BS contributed equally to the development of these ideas and writing of the manuscript. Both authors contributed to the article and approved the submitted version.

## Footnotes

1 Here, we are assuming that causal mechanisms for complex traits *can* be isolated, a premise that is far from certain. Some have proposed the “gloomy prospect” that direct, strong causes for (most) psychological traits might be out of scientific reach (Turkheimer, 2016). If those predictions prove to be correct, then all causal research will be doomed from the start. At least this time, we are choosing naïve optimism.

2 It is beyond the scope of this paper to review the central role of the PFC in executive function, although notably, the PFC is critically engaged by attentional processing, task-shifting, goal monitoring, and task performance that is generally indicative of working memory capacity. In contrast, simple storage or retention of sensory information is more generally processed elsewhere in the brain, particularly in parietal regions devoted to specific sensory domains. For a review of these systems and the specific contribution of the PFC to executive functions relevant to intelligence, see Kane and Engle (2002), Jung and Haier (2007), Colom et al. (2010), Barbey et al. (2012), Friedman and Robbins (2022).
